# Stable silencing of SNAP-25 in PC12 cells by RNA interference

**DOI:** 10.1186/1471-2202-7-9

**Published:** 2006-01-30

**Authors:** Anne L Cahill, Bruce E Herring, Aaron P Fox

**Affiliations:** 1 Department of Neurobiology, Pharmacology & Physiology, The University of Chicago, Chicago, IL, USA

## Abstract

**Background:**

SNAP-25 is a synaptic protein known to be involved in exocytosis of synaptic vesicles in neurons and of large dense-core vesicles in neuroendocrine cells. Its role in exocytosis has been studied in SNAP-25 knockout mice, in lysed synaptosomes lacking functional SNAP-25 and in cells after treatment with botulinum toxins A or E that specifically cleave SNAP-25. These studies have shown that SNAP-25 appears to be required for most but not all evoked secretion. In order to further study the role of SNAP-25 in catecholamine secretion from PC12 cells we have used the recently developed technique of RNA interference to generate PC12 cell lines with virtually undetectable levels of SNAP-25. RNA interference is the sequence-specific silencing or knockdown of gene expression triggered by the introduction of double-stranded RNA into a cell. RNA interference can be elicited in mammalian cells in a number of ways, one of which is by the expression of small hairpin RNAs from a transfected plasmid. Selection of stably transfected cell lines expressing a small hairpin RNA allows one-time characterization of the degree and specificity of gene silencing and affords a continuing source of well-characterized knockdown cells for experimentation.

**Results:**

A PC12 cell line stably transfected with a plasmid expressing an shRNA targeting SNAP-25 has been established. This SNAP-25 knockdown cell line has barely detectable levels of SNAP-25, but normal levels of other synaptic proteins. Catecholamine secretion elicited by depolarization of the SNAP-25 knockdown cells was reduced to 37% of control.

**Conclusion:**

Knockdown of SNAP-25 in PC12 cells reduces but does not eliminate evoked secretion of catecholamines. Transient expression of human SNAP-25 in the knockdown cells rescues the deficit in catecholamine secretion.

## Background

SNAP-25 along with syntaxin and synaptobrevin are the SNARE proteins known to be involved in exocytosis of synaptic vesicles in neurons and of large dense-core vesicles in neuroendocrine cells [1,2]. The role of SNAP-25 in exocytosis has been studied in a variety of different experimental preparations [3-7]. These studies have shown that SNAP-25 appears to be required for most but not all evoked secretion. In order to further study the role of SNAP-25 in catecholamine secretion from PC12 cells we have used the recently developed technique of RNA interference to generate PC12 cell lines with barely detectable levels of SNAP-25.

RNA interference is the sequence-specific "silencing" or "knockdown" of gene expression triggered by the introduction of double-stranded RNA into a cell [8-10]. In mammalian cells this double-stranded RNA must be short (<30 bp), but may be either a simple RNA duplex with two unpaired nucleotides on the 3'-ends (small interfering RNA or siRNA; [11]) or a small hairpin RNA (shRNA) [12-16]. The siRNA or shRNA may be chemically synthesized, transcribed in vitro, produced by enzymatic cleavage of long double-stranded RNA, or expressed *in situ *from a plasmid or viral vector. For cells, such as PC12 cells, where only a modest transfection efficiency can be achieved, the use of viral vectors or the establishment of cell lines stably transfected with a vector expressing an shRNA are the best approaches to RNA interference. Production of stably transfected cell lines can be the most time-consuming option, but allows one-time characterization of the degree and specificity of gene silencing and affords a continuing source of well-characterized knockdown cells for experimentation.

In the work presented here we have established and characterized a PC12 cell line stably transfected with a plasmid that expresses an shRNA specific for rat SNAP-25. These cells have <5% of normal levels of SNAP-25, but normal levels of all other synaptic proteins investigated. As expected, the SNAP-25 knockdown cells exhibited diminished catecholamine secretion in response to depolarizing stimuli.

## Results

**Figure 1:**
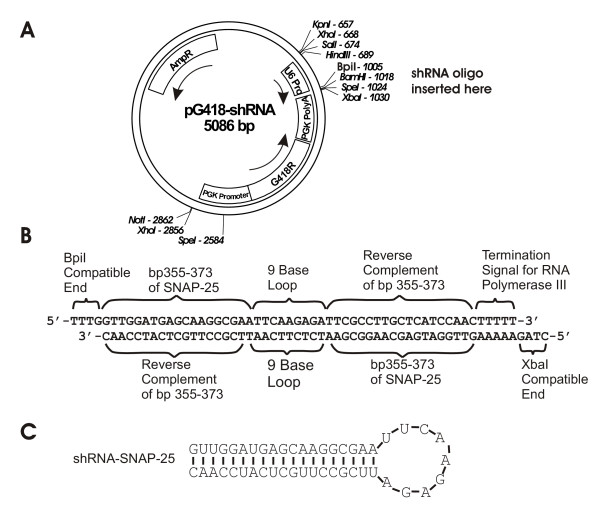
**Plasmid-based expression of shRNA-SNAP-25**. A) Plasmid map of pG418-shRNA, a plasmid designed to express shRNAs from the mouse U6 promoter. B) The two 56-base deoxyoligonucleotides used in the construction of pG418-shRNA-SNAP-25 are shown as they would be paired after annealing. The labels above and below the oligonucleotides indicate the source or the function of the nucleotides in the indicated regions. C) The predicted stem-loop structure of shRNA-SNAP-25 expressed from pG418-shRNA-SNAP-25.

**Figure 2:**
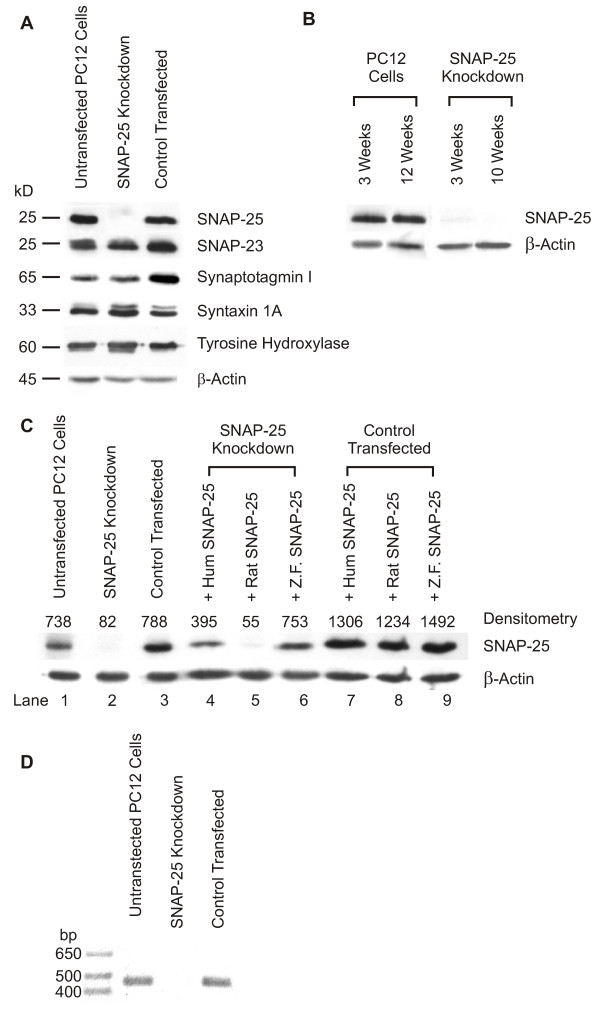
**Specific silencing of SNAP-25 by RNA interference**. A) Immunoblots were done to assess the levels of SNAP-25, SNAP-23, synaptotagmin I, syntaxin 1A, tyrosine hydroxylase and β-actin in wild type PC12 cells, SNAP-25 knockdown cells and in control transfected cells (PC12 cells stably transfected with pG418-shRNA lacking an shRNA insert). Equal amounts of protein were loaded per lane. B) The SNAP-25 phenotype is maintained for at least 10 weeks in culture in both the parent PC12 cell line and the SNAP-25 knockdown cell line. C) Human and zebrafish SNAP-25 mRNAs are resistant to RNA interference. The specificity of the SNAP-25 shRNA was demonstrated by transiently transfecting SNAP-25 knockdown cells with plasmids designed to express SNAP-25 cDNA of rat, human, or zebrafish origin. The 19 nucleotide region of rat SNAP-25 mRNA which is targeted by the SNAP-25 shRNA differs from human SNAP-25 RNA at only 2 positions and from zebrafish SNAP-25 mRNA in 4 positions. The immunoblot shows that expression of rat SNAP-25 was silenced in the SNAP-25 knockdown cells, but human and zebrafish SNAP-25 were expressed. All three SNAP-25 cDNAs appeared to be expressed in the control transfected cells in that there was more SNAP-25 in the control transfected cells than in untransfected PC12 cells. The immunoblot was stripped and reprobed for β-actin to demonstrate approximately equal amounts of protein in each sample. D) SNAP-25 mRNA is reduced in SNAP-25 knockdown cells. RT-PCR was carried out with RNA isolated from wild type PC12 cells, SNAP-25 knockdown cells and in control transfected cells. SNAP-25 mRNA was easily detected in wild type and control transfected PC12 cells, but no SNAP-25 mRNA was detected in the SNAP-25 knockdown cells in this PCR experiments. However, if more of the reverse-transcription product was used for the PCR or if more cycles were done in the PCR reaction, some SNAP-25 mRNA was detectable in the SNAP-25 knockdown cells.

A plasmid designed to express an shRNA targeting rat SNAP-25 (Figure 1) was successfully constructed and transfected into 1.25 × 10^6 ^PC12 cells. A total of 47 colonies survived selection with G418, 11 colonies were isolated and grown up, and 6 of the colonies tested positive by PCR for incorporation of pG418shRNA-SNAP-25. The stable transfection efficiency was approximately 1 cell in 50,000. All six of the stably transfected cell lines had significantly reduced levels of SNAP-25, but the extent of knockdown varied among the cell lines (data not shown). The cell line with the least (5 ± 4 % of normal SNAP-25 as determined by densitometry of 8 immunoblots) amount of SNAP-25 was chosen for further characterization and will be referred to as SNAP-25 knockdown cells (Figure 2A). The stability of the SNAP-25 knockdown phenotype was confirmed by immunoblots showing that knockdown of SNAP-25 persisted for at least 10 weekly passages of the cells (Figure 2B). In order to prevent phenotypic drift (which has been a problem with PC12 cells) the cultures were used for only 8–10 weeks before reverting to frozen stocks from an early passage. A similar number of colonies were obtained after transfection with the vector pG418shRNA lacking an shRNA insert, and one was chosen as the control stably transfected cell line. The control transfected cell line had levels of SNAP-25 comparable to the parent PC12 cells (Figure 2A). Although the SNAP-25 shRNA expressed from pG418shRNA-SNAP-25 was effective in silencing SNAP-25, it did not conform fully to the most stringent guidelines for design of effective shRNAs [17-20].

The knockdown of SNAP-25 was specific in that levels of several other proteins known to be involved in catecholamine synthesis and secretion were unchanged (Figure 2A) Specifically levels of SNAP-23, synaptotagmin I, syntaxin 1A, tyrosine hydroxylase and β-actin were unchanged in the SNAP-25 knockdown cells and in the control transfected cells. The knockdown of SNAP-25 was also very specific to rat SNAP-25 in that human and zebrafish SNAP-25 could be expressed from cDNA plasmids in the SNAP-25 knockdown cells, but expression of rat SNAP-25 from a cDNA plasmid was silenced (Figure 2C, lanes 4–6). Human and zebrafish SNAP-25 cDNA differ from the rat SNAP-25 target site by only 2 or 4 of the 19 nucleotides, but these differences were sufficient to make human and zebrafish SNAP-25 mRNA resistant to RNA interference triggered by the rat SNAP-25 shRNA. This resistance allowed the human SNAP-25 isoform to be used in rescue experiments described below [21,22]. Note that the less intense SNAP-25 bands in lanes 4 and 6 of Figure 2C do not reflect partial knockdown of human and zebrafish SNAP-25. The SNAP-25 in lanes 4 and 6 is derived solely from the ~15–20% of cells that are transiently transfected with the human and zebrafish SNAP-25 cDNA plasmids. The fact that 15–20% of the cells contain ~50% of the normal SNAP-25 means that human and zebrafish SNAP-25 is actually over-expressed (2–3 times normal) in the transiently transfected SNAP-25 knockdown cells. The more intense bands in the "Control Transfected" cell (Figure 2C, lanes 7–9) represents normal levels of SNAP-25 in all the cells plus over-expression of SNAP-25 in the 15–20% of the cells transiently transfected with the SNAP-25 expression plasmids. Densitometry of the immunoblot indicated that the amount of SNAP-25 in lanes 7–9 is approximately equal to the sum of SNAP-25 in untransfected PC12 cells (lane 1) plus the human or zebrafish SNAP-25 in lanes 4 and 6.

Under some conditions shRNAs and siRNAs can lead to repression of translation rather than degradation of specific mRNAs by RNA interference [23]. The knockdown of SNAP-25 in the cell lines described here is presumably due to RNA interference because levels of SNAP-25 mRNA (as assessed by RT-PCR) were found to be reduced in the SNAP-25 knockdown cells (Figure 2D). When a larger fraction of the reverse transcriptase reaction was used for PCR, some PCR product was obtained indicating that SNAP-25 mRNA levels are reduced but not totally eliminated. SNAP-25 mRNA levels could be quantified by real-time quantitative RT-PCR. However, knowing the extent of degradation of SNAP-25 mRNA would not enhance the utility of the SNAP-25 knockdown cell line, so this option was not pursued.

**Figure 3:**
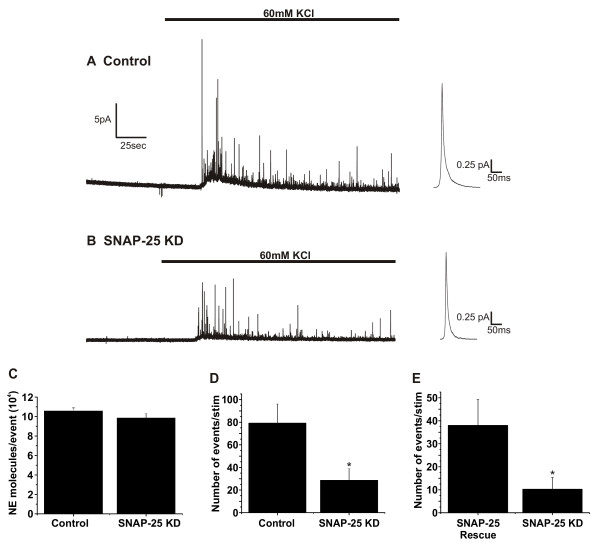
**Catecholamine secretion is reduced in SNAP-25 knockdown PC12 cells**. Representative amperometric traces are shown from a control cell stably transfected with pG418-shRNA lacking an shRNA insert (empty vector) (A) and from a SNAP-25 knockdown cell prior to and during a 2.5 min stimulation with 60 mM KCl (B). To the right of each of the traces is an averaged amperometric event shown on an expanded time scale. C) When compared to empty vector controls (n = 18) the silencing of SNAP-25 in PC12 cells (n = 18) did not alter the number of norepinephrine molecules per release event. D) Silencing of SNAP-25 resulted in a 63% reduction in the total number of exocytotic events produced following stimulation. Control transfected cells and SNAP-25 knockdown cells produced a mean ± SEM of 79 ± 17 events and 29 ± 10 events, respectively. *p < 0.03 (Student's t-test). E) Transient transfection with a human SNAP-25 expression plasmid rescued the deficit in catecholamine secretion in the SNAP-25 knockdown cells. *p < 0.03 (Student's t-test).

Amperometry experiments were carried out to determine whether knockdown of SNAP-25 affected catecholamine secretion from PC12 cells. The amperometric current recorded from a control transfected PC12 cell is shown in Figure 3A. The trace shows that application of high-K^+ ^for 2.5 minutes elicited a large number of amperometric events. Averaging every individual amperometric event during the high-K^+ ^stimulation produced the average amperometric event shown to the right (Figure 3A). Figure 3B shows similar data from a SNAP-25 knockdown PC12 cell. It too had many events, but on average fewer than did control transfected cells. Although the average amperometric event for these cells (Figure 3B, right) tended to be smaller, the difference was not significant and the amount of neurotransmitter released by both types of cells was virtually identical (Figure 3C; n = 18 control transfected cells; n = 18 knockdown cells). SNAP-25 knockdown cells produced an average of 29 release events per high-K^+ ^stimulation, while control transfected cells produced an average of 79 release events (Fig 3D). Knockdown of SNAP-25 led to a 63% reduction in the total number of exocytotic events observed. Because the area under the curve of amperometric events in control transfected and knockdown cells were virtually identical, this data suggests that SNAP-25 knockdown cells released ~37% of the neurotransmitter of that released by control transfected cells. Transient over-expression of human SNAP-25 in the SNAP-25 knockdown cells rescued the deficit in neurotransmitter release (Figure 3E). In this experiment, transfected cells were identified by co-expression of EGFP. Human SNAP-25 increased neurotransmitter release by ~3.7 fold.

## Discussion

We have used RNA interference to generate a PC12 cell line with ~5% of normal levels of the SNARE protein SNAP-25, and we have begun to characterize what effects the lack of SNAP-25 has on catecholamine secretion from these cells. RNA interference is most commonly done on a transient basis, but the use of plasmid or viral vectors designed to express shRNAs allows the generation of stable cells lines with permanently reduced levels of a specific protein [15,24,25]. Stable expression of shRNAs also allows effective suppression even of proteins that turn over slowly. Stable cell lines can be characterized as to the specificity and extent of knockdown of the targeted gene product and then used without the need to assess or make assumptions about the effectiveness of knockdown in each transient transfection. The generation of stable cell lines also affords the possibility of obtaining cells with different levels of knockdown [26,27]. Although the results reported here focus solely on the PC12 cell line with barely detectable levels of SNAP-25, several other cell lines with varying levels of SNAP-25 were generated in the course of this work. These may be useful for investigating the effects of varying levels of SNAP-25 on secretion.

The ability to rescue or reverse an RNA interference-induced effect using a version of the targeted gene that is not recognized by the siRNA or shRNA is considered to be one of the best controls for specificity in any RNA interference experiment [9,21]. In the present work we have shown that the knockdown of endogenous rat SNAP-25 in PC12 cells is specific in that human and zebrafish SNAP-25 can be expressed in the SNAP-25 knockdown cells, and transient expression of human SNAP-25 rescued the deficits in catecholamine secretion observed in the SNAP-25 knockdown cells.

The role of SNAP-25 in exocytosis previously has been studied in SNAP-25 knockout mice [3,4], in bovine chromaffin cells treated with botulinum toxins A or E to specifically cleave SNAP-25 [6,7], and in a synaptosomal membrane preparation treated with antibodies against SNAP-25 [5]. The antibody treatment reduced Ca^2+^-dependent glutamate release by 60–70%, but it was not clear whether the residual secretion was due to SNAP-25 that had escaped inactivation by the antibody or due to a SNAP-25-independent mechanism. Cleavage of bovine chromaffin cell SNAP-25 by botulinum toxin E almost completely inhibited the secretion of catecholamines elicited by intracellular uncaging of chelated Ca^2+ ^[6]. In contrast botulinum toxin A only partially inhibited catecholamine secretion [6], and this inhibition could be partially overcome by higher intracellular levels of Ca^2+ ^[7]. These studies provided strong evidence for a crucial role of SNAP-25 in exocytosis, but neither antibodies not botulinum toxins can produce an experimental preparation that truly lacks SNAP-25 as do homozygous SNAP-25 knockout mice [3]. In neurons cultured from embryonic SNAP-25 knockout mice, depolarization-induced exocytosis was not detectable while spontaneous neurotransmission persisted. In chromaffin cells isolated from embryonic SNAP-25 knockout mice, the fast burst of catecholamine secretion in response to uncaging of chelated Ca^2+ ^was abolished while a more prolonged elevation of intracellular Ca^2+ ^obtained by infusion of 10 μM Ca^2+ ^through a patch pipet reduced the number of amperometric spikes of catecholamine secretion in SNAP-25 knockout cells to about 33% of that in control cells [4]. This reduction in sustained secretion is very similar to the reduction in number of amperometric spikes measured in our SNAP-25 knockdown PC12 cells (37% of control). The slow kinetics of bath application of high K+ did not allow us to evaluate the effect of SNAP-25 knockdown on the burst components of exocytosis. Also similar to the findings in this study, the kinetic parameters of the amperometric spikes in SNAP-25 knockout chromaffin cells were similar to those found in wild-type cells [4].

There has been one other report of SNAP-25 knockdown by RNA interference [28]. In this study a plasmid expressing a SNAP-25 shRNA was used to transiently knockdown SNAP-25 in cultured hippocampal neurons, but neurotransmitter release from these neurons was not examined.

The catecholamine secretion that persists in the SNAP-25 knockdown cells may be mediated by SNAP-25 homologs such as SNAP-23. We did not observe any compensatory increase in SNAP-23 levels in the SNAP-25 knockdown cells, but the possible role of SNAP-23 or other SNARE proteins could be investigated by generating cell lines expressing two or more shRNAs targeting distinct proteins. It has been shown that it is possible to use RNA interference to knockdown more than one gene product [29,30], and we have preliminary evidence that PC12 cells stably transfected with at least two plasmids can be generated at a reasonable frequency. The ability to specifically reduce levels of SNAP-25 and other constituent proteins of the SNARE complex in PC12 cells will provide a unique opportunity to study the roles of each of these proteins in exocytosis.

## Conclusion

A PC12 cell line stably transfected with a plasmid expressing an shRNA targeting SNAP-25 has been established. This SNAP-25 knockdown cell line has ~5% of normal levels of SNAP-25, but normal levels of other synaptic proteins. Catecholamine secretion elicited by depolarization of the SNAP-25 knockdown cells was reduced to 37% of control, but was restored by transient expression of human SNAP-25. The combination of RNAi and subsequent rescue with a gene from a different species in a cell line provides a viable method for the study of synaptic proteins that would otherwise be lethal in animals.

## Methods

### Plasmid construction

When this project was started, plasmids for the expression of shRNAs had been described in the literature [12-16,31], but were not yet commercially available. In addition none of the described plasmids contained drug-resistance genes allowing selection of stably transfected cells. Therefore, an shRNA expression plasmid containing the mouse U6 RNA polymerase III promoter and a selection cassette to confer resistance to G418 was constructed. The promoter from the mouse U6 RNA polymerase III gene was cloned by PCR using mouse genomic DNA as the template and primers designed from the published sequence of the mouse U6 gene [GenBank:X06980]. The PCR primers were also designed to incorporate Hind III, Bpi I and Bam HI restriction sites on the ends of the PCR product. The U6 promoter PCR product was digested with Hind III and BamHI and ligated into pBSII cut with these same enzymes. The U6 promoter region was sequenced to ensure no mistakes had been introduced during the PCR cloning. The G418-resistance cassette from pPNT [32] was then sublconed into the U6-pBSII plasmid to yield pG418shRNA (Figure 1A).

A target region for silencing rat SNAP-25 was selected from a region of the cDNA that is common to both SNAP-25a and SNAP-25b so that both isoforms would be targeted. The selected site (GTTGGATGAGCAAGGCGAA, bp 355–373 of [GenBank:NM_030991]) conformed to many of the guidelines for effective shRNAs: 52% G+C content; no regions with >9 G/C; no runs of >3 of any nucleotide; G on the 5'-end; A on the 3'-end; located >100 nucleotides from the start codon [19]; but did not fully meet the more advanced criteria for effective shRNAs that are based on thermodynamic properties of the target sequence [17,18]. The loop sequence chosen for the shRNA (TTCAAGAGA) was suggested by Brummelkamp et al. [15]. Complementary deoxyoligonucleotides containing the shRNA target sequence, the loop sequence, the reverse complement of the target sequence, an RNA polymerase III termination sequence, and restriction-site compatible overhangs were designed as shown in Figure 1B and were purchased from IDT (Coralville, IA). The deoxyoligonucleotides were annealed and ligated into pG418shRNA to yield pG418shRNA-SNAP-25. The predicted shRNA expressed by this plasmid is shown in Fig 1C.

### Transfection and selection of stably transfected PC12 cells

PC12 cells (1.25 × 10^6^) [33] were transfected with 4 μg of either pG418shRNA-SNAP-25 or pG418shRNA using Lipofectamine 2000. One day after transfection the cells were replated at a lower density and selected with 100 μg/ml G418 for 26 days until discrete colonies were formed. PC12 cells grow slowly with a doubling time of about 3 days, hence the relatively long time before G418 resistant colonies were formed. Individual colonies were isolated, grown up and tested by PCR for the incorporation of the plasmid into genomic DNA.

### Immunoblotting

Levels of SNAP-25, SNAP-23, syntaxin 1A, synaptotagmin I, tyrosine hydroxylase and β-actin in the cell lines were assessed by immunoblotting with the following antibodies: SNAP-25 (ANR-001, Alomone), SNAP-23 (DS-19; Sigma), syntaxin 1A (#573831, Calbiochem), synaptotagmin I (mAb48; Developmental Systems Hybridoma Bank, University of Iowa), tyrosine hydroxylase (#657010, Calbiochem), β-actin (JLA20; Developmental Systems Hybridoma Bank, University of Iowa), and horseradish peroxidase-labeled anti-mouse or anti-rabbit IgG (Jackson ImmunoResearch). ECL Advance reagents (Amersham/GE Healthcare) were used for detection of the horseradish peroxidase-labeled secondary antibodies.

### RT-PCR

Total RNA was isolated using Trizol (Invitrogen) and the RNA was reverse-transcribed using Superscript II (Invitrogen) and random hexamers. PCR was carried out using first-strand cDNA obtained by reverse transcription and primers that span exon junctions of the rat SNAP-25 gene (Exons 3–4: CGAAGAGAGTAAAGATGCTGGC; bp 316–338 of rat SNAP-25 [GenBank:NM_030991]; and Exons 7–8 GTTTTGTTGGAGTCAGCCTTCT; reverse complement of bp 755–777). The use of primers that span exon junctions assures that the PCR product is amplified only from cDNA and not genomic DNA.

### Expression of SNAP-25 Isoforms resistant to RNA interference

Plasmids for expression of rat, human and zebrafish SNAP-25 were obtained from ATCC (IMAGE Clone IDs: 7314318, 3867544, and 6996354) and transfected into the SNAP-25 knockdown and the control transfected cells using Lipofectamine 2000. SNAP-25 levels were assessed by immunoblotting. Human, rat and zebrafish SNAP-25 were all recognized by the anti-SNAP-25 antibody. In experiments designed to assess whether human SNAP-25 could rescue the deficit in catecholamine secretion, pEGFP-N1 was co-transfected with the human SNAP-25 IMAGE clone to allow identification of transiently transfected cells.

### Catecholamine secretion and amperometry

Carbon fiber electrodes were fabricated and prepared for recording as previously described [34]. On the day of the experiment all cells were loaded with catecholamine transmitter by 2–4 hr incubations in culture media containing 2 mM norepinephrine prior to recording. The electrode was pressed gently against the cell during the recording for the highest collection efficiency possible [35,36]. The electrode was held at + 700 mV *vs *ground using an NPI VA-10 amplifier to oxidize catecholamine transmitter. The amperometric signal was low-pass filtered at 2 kHz (8-pole Bessel; Warner Instruments, Hamden, CT, USA). A 16-bit analog-to-digital converter (National Instruments Corp., Austin, TX) was interfaced with custom data acquisition software. The amperometric signal was acquired at 10 kHz and stored on a personal computer. Amperometric spike features and kinetic parameters were analyzed using a series of macros written in Igor Pro (Wavemetrics Inc.) kindly supplied by Dr. Eugene Mosharov (Columbia University). The detection threshold for an event was set at 4 – 5 times the baseline root mean squared noise, and the spikes were automatically detected. Amperometric data was obtained from 66 cells.

### Recording solutions and stimulation protocol

Recordings were made from adherent cells that were under constant perfusion (flow rate of 1.0 ml/min, and a chamber volume of ~150 μl). The wash solution had the following standard composition: 145 mM NaCl, 2.0 mM KCl, 10 mM HEPES, 2 mM CaCl_2 _and 1.0 mM MgCl_2_. High potassium (high-K^+^) solutions contained 82 mM NaCl, 60 mM KCl, 10 mM HEPES, 2 mM CaCl_2 _and 1.0 mM MgCl_2_. The solutions were adjusted to a final pH of 7.3 and an osmolarity of 300 ± 3 mOsm/kg. All experiments were performed at ambient temperature (24° ± 2°C). Cells were initially perfused with wash solution for at least 1 min prior to being stimulated with High-K^+ ^solution for 2.5 mins. Cells were subjected to only a single stimulation.

## List of abbreviations

shRNA: small hairpin RNA

siRNA: small interfering RNA

SNAP-25: synaptosome-associated protein of 25 kD

SNAP-23: synaptosome-associated protein of 23 kD

SNARE: soluble N-ethylmaleimide sensitive factor attachment protein receptor

RT-PCR: reverse transcription-polymerase chain reaction

## Authors' contributions

ALC conceived of the study, participated in its design, constructed the plasmids, generated the stably transfected cell lines, carried out the immunoblot and RT-PCR experiments, and drafted the manuscript. BH carried out the amperometry experiments, analyzed the amperometric data and helped draft the manuscript. APF participated in the design of the study, the acquisition and analysis of the amperometry data, and the writing of the manuscript. All authors read and approved the final manuscript.
